# The Effect of *G0S2* Gene Knockout on the Proliferation, Apoptosis, and Differentiation of Chicken Preadipocytes

**DOI:** 10.3390/ani15070951

**Published:** 2025-03-26

**Authors:** Yantao Li, Boyu Wang, Zhaochuan Wang, Jintian Wen, Tianle Zhou, Jiahao Tang, Zhenhui Li

**Affiliations:** 1 State Key Laboratory of Swine and Poultry Breeding Industry, Guangdong Provincial Key Lab of Agro-Animal Genomics and Molecular Breeding, College of Animal Science, South China Agricultural University, Guangzhou 510642, China; 20222024011@stu.scau.edu.cn (Y.L.); bywang@stu.scau.edu.cn (B.W.); zuisc@stu.scau.edu.cn (Z.W.); wenjintian@stu.scau.edu.cn (J.W.); tianle_zhou@outlook.com (T.Z.); 18312735239@163.com (J.T.); 2 Key Laboratory of Chicken Genetics, Breeding and Reproduction, Ministry of Agriculture and Rural Affair, South China Agricultural University, Guangzhou 510642, China

**Keywords:** lipocyte, *G0S2*, function, CRISPR/Cas9, RNA-seq

## Abstract

The G0/G1 switch gene 2 (*G0S2*) has been shown to be involved in cell proliferation, apoptosis, and differentiation in mammals. However, its function in poultry is not fully understood, especially in preadipocytes of chickens. This study aimed to establish a *G0S2* knockout preadipocyte cell line in chickens through CRISPR/Cas9 technology and to thoroughly investigate the impact of *G0S2* on chicken preadipocyte proliferation, apoptosis, and differentiation. To explore the involvement of *G0S2* in chicken preadipocyte growth and development, transcriptome sequencing was performed. The results demonstrated that *G0S2* was successfully deleted using the CRISPR/Cas9 system. *G0S2* knockout significantly inhibited the differentiation of chicken preadipocytes while promoting their proliferation. Additionally, although *G0S2* knockout exhibited a pro-apoptotic effect, it was relatively mild, primarily reflected in an increased proportion of early apoptotic cells. *G0S2* deletion significantly affected the expression of important genes related to lipid metabolism, cell cycle control, and signaling pathways, based on transcriptomic analysis. In conclusion, our findings suggest that *G0S2* performs a critical role in regulating chicken preadipocyte differentiation, proliferation, and apoptosis. This research offers valuable new insights into the molecular mechanisms and control of *G0S2* in the growth and development of chicken preadipocytes.

## Simple Summary

This study aimed to investigate the function of the *G0S2* gene in chicken preadipocytes using a *G0S2* knockout cell line created with CRISPR/Cas9 technology. The results demonstrated that *G0S2* knockout promoted the proliferation and inhibited the differentiation of chicken preadipocytes. Additionally, although *G0S2* knockout exhibited a pro-apoptotic effect, it was relatively mild, primarily reflected in an increased proportion of early apoptotic cells. Transcriptome analysis indicated significant changes in key genes related to lipid metabolism, the cell cycle, and signal transduction pathways. The results of this study are beneficial for revealing the function of the *G0S2* gene in chicken preadipocytes and for deeply understanding the genetic mechanism of chicken adipose tissue growth and development.

## 1. Introduction

The poultry industry has significantly improved the growth rate and feed conversion efficiency of broilers via the use of high-energy diets and genetic selection of commercial chickens [1]. Nonetheless, high abdominal fat deposition—more than 85% of which serves no functional purpose in the body—is frequently associated with the fast development of broilers [2]. In addition to decreasing feed efficiency and affecting laying hens’ ability to reproduce, excessive abdominal fat deposition has a detrimental impact on the slaughter process and may lead to environmental contamination [2,3,4]. Additionally, more abdominal fat raises the amount of fat in chicken flesh, which increases the risk of cardiovascular illnesses in people [5].

Both the size and number of adipocytes increase, contributing to the growth of adipose tissue [6]. Preadipocyte differentiation mainly controls adipocyte size, while mechanisms such as preadipocyte proliferation, differentiation, and apoptosis influence the number of adipocytes [7,8,9,10,11]. Therefore, comprehensive research on the growth, development, and molecular regulation of adipocytes in broilers has become a central focus in poultry production.

In recent years, the G0/G1 switch gene 2 (*G0S2*) has emerged as a research hotspot, with its involvement in cell proliferation, differentiation, and adipocyte becoming clearer. *G0S2* was first discovered as a cell cycle-related gene, with expression tightly linked to the transition of cells from the quiescent phase (G0) to the proliferative phase (G1) [12,13]. Subsequent research has shown that *G0S2* is also important in lipid metabolism. *G0S2* specifically inhibits adipose triglyceride lipase’s (ATGL) lipolytic activity, controlling lipid metabolic balance in adipocytes [14]. Additionally, *G0S2* is a direct target of *PPARγ*, and treatment with the *PPARγ* agonist rosiglitazone notably increases *G0S2* protein expression in mouse adipocytes [14,15]. *G0S2* also has a role in regulating apoptosis. Previous research has demonstrated that *G0S2* interacts with *Bcl-2*, promoting apoptosis in tumor cells [16]. However, another study showed that *G0S2* protects endothelial cells from serum deprivation and hydrogen peroxide-induced apoptosis [17].

Although the functions of *G0S2* in mammals have been extensively studied, research on its role in poultry is not fully understood and is primarily focused on lipid metabolism. For example, Shin et al. demonstrated that overexpression of *G0S2* in transgenic quail inhibits lipolysis during feed restriction, highlighting its important role in poultry lipid metabolism [18]. Park et al. further found that disruption of the *G0S2* gene in chickens reduces abdominal fat deposition and alters the fatty acid composition, providing additional evidence for the critical role of *G0S2* in poultry fat metabolism [19]. In summary, research on *G0S2* in poultry is relatively scarce and mainly concentrated on lipid metabolism. Therefore, further exploration of the role and regulatory mechanisms of *G0S2* in chicken preadipocytes is of significant importance.

In recent years, the rapid development of gene editing technologies has provided more precise tools for exploring gene functions. As an emerging gene editing tool, CRISPR/Cas9 has been widely applied in functional genomics research due to its efficiency, simplicity, and low cost [20,21]. Additionally, Homology-Mediated End Joining (HMEJ) [22], Homology-Independent Targeting Integration (HITI) [23], and Homology-Directed Repair (HDR) [24] are three important gene editing strategies. Through a comparison of these strategies, HMEJ has been proven to be a robust and effective method for gene knock-in in chicken primordial germ cells (PGC) [25]. To further evaluate the effectiveness of these strategies in different cell types, subsequent experiments will focus on a comparative analysis of the three strategies in chicken preadipocytes. When combined with CRISPR/Cas9, these strategies can achieve more precise and efficient gene editing, providing a reliable technical foundation for constructing knockout cell lines and gene function research.

Building on the previously mentioned background, this study utilized CRISPR/Cas9 in combination with the HMEJ donor integration method to generate a G0S2 knockout preadipocyte cell line in chickens. It then carefully analyzed the impact of *G0S2* on the cell cycle, cell proliferation, differentiation, and apoptosis of chicken preadipocytes. The findings aim to clarify the function of *G0S2* in the growth and development of chicken preadipocytes, contributing valuable insights into the molecular control of poultry fat metabolism and the optimization of fat accumulation.

## 2. Materials and Methods

### 2.1. Cell Culture and Differentiation

Northeast Agricultural University kindly donated the ICP2 cell line (Harbin, China), which was used in our study [26]. The cells were cultured at 37 °C in an environment with 5% CO_2_. They were kept in full DMEM/F12 media (Gibco, Thermo Fisher Scientific, Grand Island, NY, USA), supplemented with 10% fetal bovine serum (FBS; VivaCell, Shanghai, China, catalog number: C04002-500) and 1% penicillin-streptomycin (Gibco, Thermo Fisher Scientific, Grand Island, NY, USA). The ICP2 cells were subjected to differentiation induction media (DMEM/F12 supplemented with 10% FBS and 260 μM sodium oleate; KunChuang, Xi’an, China) when they had reached 60–80% confluence. Every 24 h, the differentiation medium was changed.

### 2.2. Establishment of G0S2 Knockout Chicken Preadipocyte Cell Line

Three highly specific sgRNAs targeting the *G0S2* gene of the chicken were designed using the website http://crispor.tefor.net/ (accessed on 7 March 2024). The corresponding oligonucleotide sequences are listed in Table 1.

The BbsI restriction enzyme (built by GeneScript) was used to introduce the sgRNAs into the PX459 plasmid. The Cas9/gRNA plasmid was added to the growth media once the ICP2 cells had reached 60–80% confluence. After seven days, cells that successfully transfected were chosen using puromycin (1 µg/mL). Following the extraction of genomic DNA, T7E1 nuclease digestion and Sanger sequencing of TA-cloned products were used to evaluate the cutting effectiveness of the sgRNA. Sanger sequencing was performed on 10 samples per group, with three independent experiments. Based on the highest editing efficiency, donor plasmids for various integration strategies (HMEJ, HDR, HITI) were designed and synthesized by GeneScript. Cas9/gRNA plasmids and donor plasmids were co-transfected into ICP2 cells. After another 7-day puromycin selection, the cells were cultured for an additional 7 days in a regular medium. Using *EGFP* (Enhanced Green Fluorescent Protein) as a selection marker, flow cytometry was used to assess the various techniques’ integration efficiency. Lastly, ICP2 cells were co-transfected with the HMEJ donor plasmid and the sgRNA with the best targeting efficiency. Using flow cytometry, positive cells were sorted. The effective *G0S2* deletion in the chicken preadipocyte cell line was confirmed by RT-qPCR and transcriptome analysis of the *G0S2* expression levels in the sorted cells.

These *G0S2* knockout cells are designated as *G0S2*-KO cells, while the ICP2 cells are referred to as WT cells.

### 2.3. Cell Proliferation Assay

The EdU Apollo 567 kit (Ribobio, Guangzhou, China) and the CCK-8 assay (NCM Biotech, Suzhou, China) were used to measure cell proliferation. In 96-well plates, *G0S2*-KO and WT cells were planted at a density of 3 × 10^4^ cells per well. Each well received 10 μL of CCK-8 solution after 24 h, and the cells were then incubated for an extra hour at 37 °C. At 450 nm, absorbance was measured. The cells were fixed with 4% paraformaldehyde for 30 min, treated with 50 μM EdU for 2 h, and then rinsed with PBS in order to perform the EdU analysis. After 30 min of 1× Apollo solution staining, the cells were subjected to a further 30 min of Hoechst 33342 staining. Cell quantification was carried out using ImageJ (version 1.54g) after fluorescent pictures were taken.

### 2.4. Cell Cycle Assay

A cell cycle detection kit (Coolaber, Beijing, China) was used to perform cell cycle analysis. Cells were first trypsinized and then thrice washed with ice-cold 1× PBS. The cell pellet was gathered following centrifugation, and the supernatant was disposed of. To enable fixation, the cells were carefully reconstituted in 1 milliliter of ice-cold 70% ethanol, and the suspension was then incubated for 4 h at 4 °C. Following fixing, the cells underwent another centrifugation, and the supernatant was disposed of. The cells were rinsed twice with 1× PBS to get rid of any remaining ethanol, and 500 μL of PI/RNase staining buffer was used to stain each sample. After that, the cells were reconstituted and incubated for 30 min at 37 °C in the dark. Using flow cytometry, the distribution of the cell cycle was examined (Agilent Technologies, Santa Clara, CA, USA).

### 2.5. Cell Apoptosis Assay

An Annexin V-APC/PI detection kit (Leagene, Hangzhou, China) was used to quantify apoptosis. The steps were as follows: For cell counting, the trypsinized cells were resuspended in PBS after being trypsinized without EDTA. Following a 5-min centrifugation of 5–10 × 10^4^ cells at 1000× *g*, the pellet was resuspended in 500 μL of 1× Annexin V solution, and the supernatant was disposed of. Following a 15-min dark incubation period at room temperature (20–25 °C), 5 μL of Annexin V-APC and 5 μL of PI staining solution were applied to the cells. Flow cytometry was then used to measure apoptosis.

### 2.6. Oil Red O Staining and Extraction Assay

Following two PBS washes, differentiated *G0S2*-KO and WT cells were preserved for 30 min in 4% paraformaldehyde. Following fixation, the cells were washed with distilled water and stained for 15 min using Oil Red O, which is a 3:2 solution of Oil Red O and water. After 20 s of immersion in 60% isopropanol, the cells were cleaned once more and observed under a microscope (Leica, Wetzlar, Germany). Additionally, 100% isopropanol was used to elute the stain from the Oil Red O, and the lipid content was measured at 510 nm using a multifunctional plate reader (Gene Company Limited, Hong Kong, China).

### 2.7. RNA Extraction and RT-qPCR

TRIzol reagent (Invitrogen, Grand Island, NY, USA) was used to extract total RNA from cells in accordance with the manufacturer’s instructions. Reverse transcriptase (Novizan, Nanjing, China) was used to synthesize cDNA, and β-actin served as the internal control to bring gene expression levels back to normal. Predenaturation at 95 °C for 5 min, denaturation at 95 °C for 30 s, annealing at 58 °C for 30 s, extension at 72 °C for 1 min, 40 cycles, and a final extension at 72 °C for 10 min were the conditions under which the synthesized cDNA was utilized as a template for PCR amplification. The Applied Biosystems QuantStudio 5 equipment was used for RT-qPCR, and the ΔΔCt method was used to assess gene expression levels for relative quantification. Table 2 lists the primer sequences used for qPCR. Primers were created using Primer 5.0 software based on NCBI gene sequences.

### 2.8. Western Blotting Assay

Well-conditioned *G0S2*-KO and WT cells were incubated with RIPA buffer containing protease inhibitors (protease inhibitors:RIPA = 1:100, Solarbio, Guangzhou, China) on ice for 15 min to fully lyse the cells. Subsequently, the lysates were centrifuged at 12,000× *g* for 10 min at 4 °C, and the supernatant was collected. Proteins were separated using 12% SDS-PAGE and transferred onto nitrocellulose membranes (Whatman, Maidstone, UK), blocked with 5% skim milk powder for 1 h, and then incubated with primary antibody solution overnight at 4 °C. The membranes were then washed three times with TBST solution (Beyotime, Haimen, Jiangsu, China), each for 5 min, followed by incubation with secondary antibody solution at room temperature for 60 min. Western blotting results were analyzed using the Odyssey Fc system (LI-COR, Lincoln, NE, USA). Antibody information is as follows: activated caspase-3 rabbit polyclonal antibody (19677-1-AP; Proteintech Group, Wuhan, China; 1:1000) and Bate Actin mouse polyclonal antibody (66009-1-Ig; Proteintech Group, Wuhan, China; 1:1000).

### 2.9. RNA-Seq

The quality and concentration of total RNA extracted from G0S2-KO and WT cells were assessed using the Agilent 2100 Bioanalyzer (Agilent Technologies, Santa Clara, CA, USA). mRNA was enriched with oligo(dT) magnetic beads, followed by cDNA synthesis using reverse transcriptase. The library was then purified, end-repaired, A-tailed, and ligated with adapters. AMPure XP beads were used to select appropriate fragments, followed by PCR amplification to construct the library. The quality of the library and insert size were verified using the Qubit 2.0 Fluorometer and the Agilent 2100 Bioanalyzer. Paired-end sequencing with 150 bp reads was performed using Illumina sequencing. To ensure high-quality data for downstream analysis, adapter sequences, low-quality reads, and sequences containing Ns were removed during quality control. Differential expression analysis (DEGs) was carried out using DESeq2 (version 1.16.1) software on the clean data, which was aligned with the Gallus gallus reference genome (Ensembl version: GRCg6a, release 106) using HISAT2.

### 2.10. Functional and Enrichment Analysis

The clusterProfiler program (version 3.8.1) was used to perform Kyoto Encyclopedia of Genes and Genomes (KEGG) pathway enrichment studies and Gene Ontology (GO) functional enrichment analyses.

### 2.11. Statistical Analysis

GraphPad Prism 9.5.1 was used for statistical analysis. The mean ± standard deviation (SD) is used to display the data. The differences between the *G0S2*-KO and WT groups were compared using a Student’s *t*-test. Statistical significance was defined as a *p*-value < 0.05, whilst no significant difference was indicated by a *p*-value > 0.05.

## 3. Results

### 3.1. Comparison of HITI, HMEJ, and HDR Donor Integration Efficiency and Construction of G0S2 Knockout Preadipocyte Cell Line in Chickens

**Figure 1:**
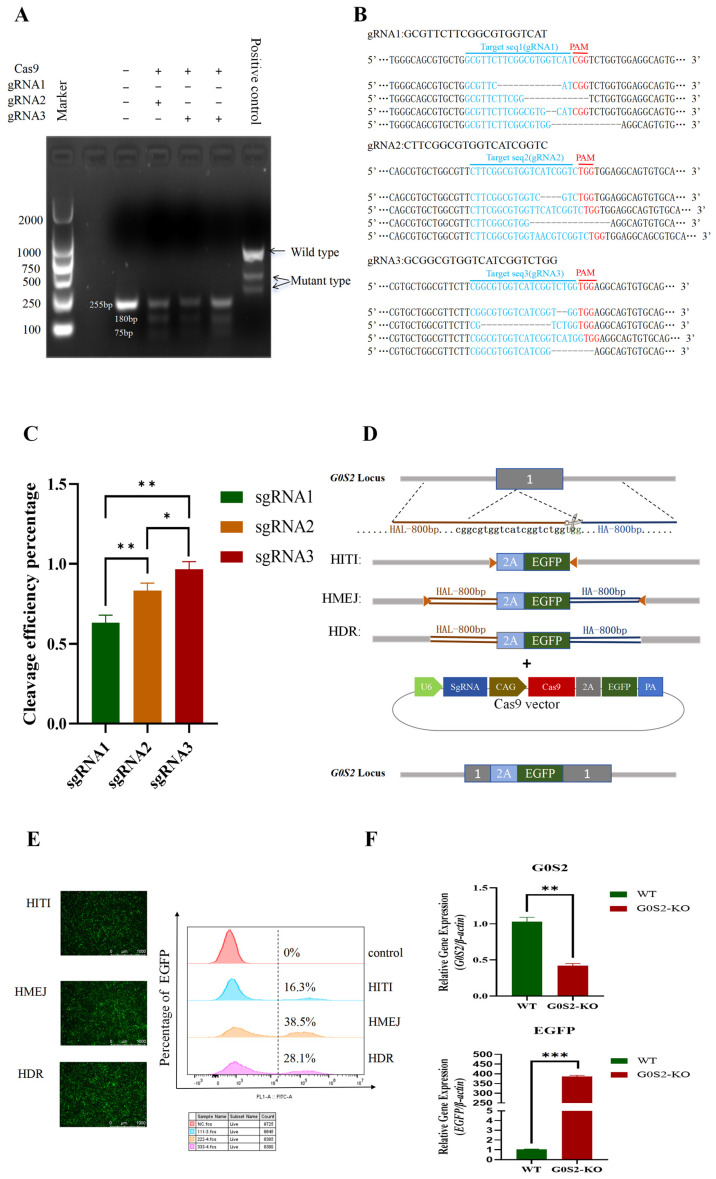
Comparison of HITI, HMEJ, and HDR donor integration efficiency and the construction of a *G0S2* knockout preadipocyte cell line in chickens. (**A**) gRNA cleavage activity assay. (**B**) Sanger sequencing results and gene editing type analysis. (**C**) gRNA cleavage activity detection by Sanger sequencing. (**D**) The diagram illustrates the design of the three donor plasmids, where the orange triangles represent the single guide RNAs (sgRNAs), and HAL-800bp and HA-800bp represent the left and right homologous arms, respectively. (**E**) Comparison of the integration efficiency of three donor plasmids. (**F**) Detection of *G0S2* and *EGFP* mRNA expression levels in WT and *G0S2*-KO cell lines. All data represent three independent experiments and are expressed as mean ± SD (* *p* < 0.05, ** *p* < 0.01, *** *p* < 0.001).

First, the cleavage efficiency of three sgRNAs was compared. T7E1 cleavage analysis revealed smaller mutation bands in the experimental group transfected with sgRNA plasmids, compared to the control group. Before cleavage, the original band size was 255 bp, while after cleavage, two smaller bands were observed at 180 bp and 75 bp. The sgRNA3 group exhibited the highest mutation band content (Figure 1A). Further TA cloning and Sanger sequencing analysis revealed the cleavage efficiencies of the three sgRNAs: 63% (sgRNA1), 80% (sgRNA2), and 96% (sgRNA3) (Figure 1B,C). Therefore, sgRNA3, which exhibited the highest cleavage efficiency, was chosen for subsequent experiments. To construct a pure *G0S2* knockout cell line and compare the integration efficiency of different strategies in chicken preadipocytes, three donor plasmids were designed and synthesized by Genescript (Figure 1D). These included HITI, HMEJ, and HDR. The results showed that the integration efficiency was the highest for the HMEJ strategy (38.5%), followed by HDR (28.1%). The lowest efficiency was observed with HITI (16.3%) (Figure 1D). Based on these results, the HMEJ strategy combined with CRISPR/Cas9 was employed to construct a *G0S2* knockout chicken preadipocyte cell line.RT-qPCR results indicated that *G0S2* mRNA expression was significantly lower in the *G0S2* knockout cell line compared to WT cells. In contrast, *EGFP* mRNA expression was significantly higher (Figure 1F). This confirmed the effective knockout of *G0S2* in the knockout cell line.

### 3.2. G0S2 Knockout Promotes the Proliferation of Chicken Preadipocytes

**Figure 2:**
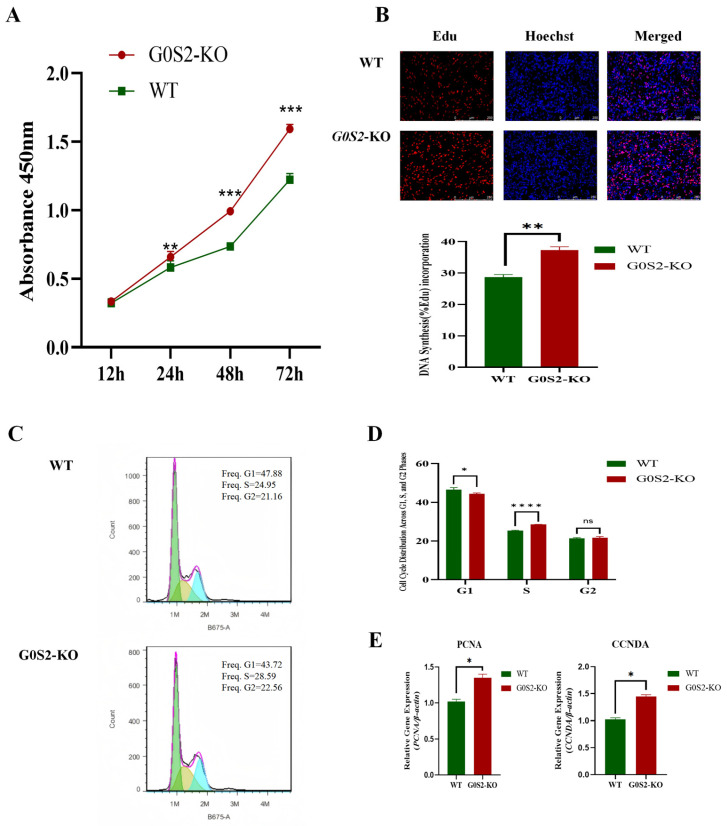
*G0S2* knockout enhances chicken preadipocyte proliferation. (**A**) *G0S2* knockout’s effect on proliferation rate. (**B**) *G0S2* knockout’s impact on DNA synthesis. (**C**) *G0S2* knockout’s effect on cell cycle distribution. (**D**) Cell Cycle Distribution Across G1, S, and G2 Phases. (**E**) Quantitative expression of proliferation-related genes in *G0S2*-KO and WT groups. All data represent the results of three independent experiments and are presented as the mean ± SD (* *p* < 0.05, ** *p* < 0.01, *** *p* < 0.001, **** *p* < 0.0001, ns *p* > 0.05).

Initially, the impact of *G0S2* deletion on the proliferation of chicken preadipocytes was evaluated using the CCK-8 assay. At 24, 48, and 72 h, *G0S2*-KO cells exhibited a significantly higher rate of proliferation compared to WT cells (*p* < 0.05, Figure 2A). This increased proliferation was further explored with EdU labeling, revealing a higher DNA synthesis rate in *G0S2*-KO cells at 48 h (*p* < 0.01, Figure 2B). The effects of *G0S2* knockout on the cell cycle were assessed by flow cytometry and PI labeling. *G0S2*-KO cells showed a significantly higher percentage of cells in the S phase (*p* < 0.01, Figure 2C) and a reduced percentage in the G1 phase (*p* < 0.05) compared to WT cells.

### 3.3. G0S2 Knockout Mildly Promotes Early Apoptosis in Chicken Preadipocytes

**Figure 3:**
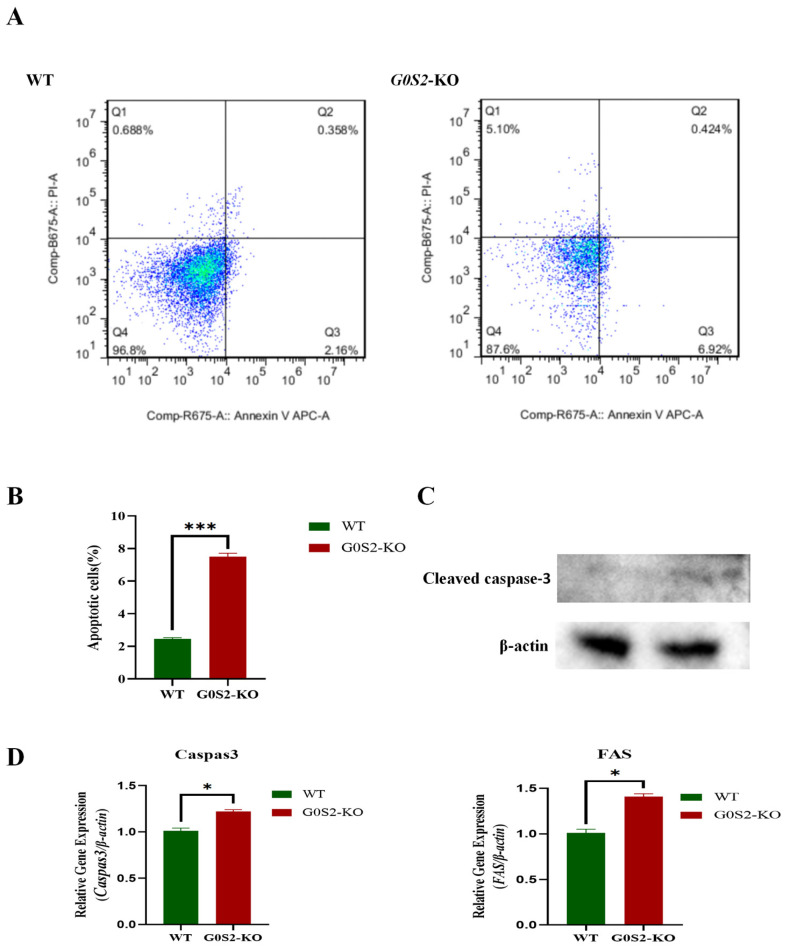
*G0S2* knockout induces apoptosis in chicken preadipocytes. (**A**) Apoptotic cell proportions at various stages in WT and *G0S2*-KO cell lines. (**B**) Impact of *G0S2* deletion on cell apoptosis in chicken preadipocytes. (**C**) The effect of *G0S2* knockout on the cleavage of caspase-3 protein. (**D**) Expression of apoptosis-related genes in the *G0S2*-KO and WT groups. All data represent the results of three independent experiments and are presented as the mean ± SD (* *p* < 0.05, *** *p* < 0.001).

The impact of *G0S2* deletion on apoptosis in chicken preadipocytes was assessed using an Annexin V-APC/PI apoptosis assay. The results showed that, after 48 h, the proportion of apoptotic cells in *G0S2* knockout cells was significantly higher than that in wild-type (WT) cells (*p* < 0.05, Figure 3A,B), although the overall apoptotic effect was relatively minor, with the main differences observed in early apoptotic cells. Further qPCR analysis revealed that *G0S2* knockout significantly upregulated the expression levels of key apoptosis-related genes, such as *caspase-3* and *Fas*. In addition, to further verify the effect of *G0S2* deficiency on apoptosis in chicken preadipocytes, Western blot analysis was performed to detect the cleavage level of caspase-3. The results showed that the cleavage level of caspase-3 was increased in *G0S2* knockout cells compared to WT cells, although the increase was not particularly significant. These findings suggest that *G0S2* knockout promotes apoptosis in chicken preadipocytes; however, the pro-apoptotic effect remains relatively mild, primarily characterized by an increased proportion of early apoptotic cells, with minimal impact on late apoptosis.

### 3.4. G0S2 Knockout Inhibits Differentiation of Chicken Preadipocytes

**Figure 4:**
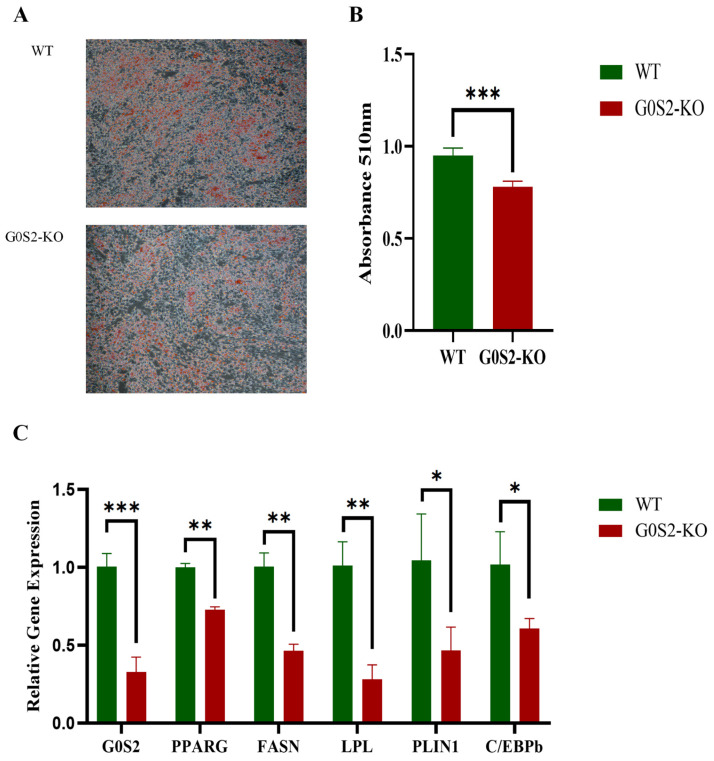
*G0S2* knockout suppresses differentiation of chicken preadipocytes. (**A**) Oil Red O staining. (**B**) Oil Red O extraction. (**C**) *G0S2* knockout affects the expression of differentiation marker genes in chicken preadipocytes. All data represent the results of three independent experiments and are presented as the mean ± SD (* *p* < 0.05, ** *p* < 0.01, *** *p* < 0.001).

Oil Red O staining and lipid extraction tests were conducted to investigate the effects of *G0S2* deletion on chicken preadipocyte differentiation. The results showed that, compared to WT cells, intracellular lipid droplet accumulation was significantly reduced in *G0S2*-KO cells during 72 h of differentiation (Figure 4A). Additionally, *G0S2*-KO cells had considerably lower lipid content than WT cells (*p* < 0.01, Figure 4B). RT-qPCR confirmed these findings. After 72 h of differentiation, *G0S2*-KO cells exhibited a marked decrease in mRNA expression of key genes related to adipogenesis, for example, *PPARγ* (Peroxisome proliferator-activated receptor gamma), *FASN* (Fatty acid synthase), *LPL* (Lipoprotein lipase), *PLIN1* (Perilipin 1), and *C/EBPβ* (CCAAT/enhancer-binding protein beta), when assessed against WT cells (*p* < 0.05 or *p* < 0.01, Figure 4C).

### 3.5. Transcriptome Sequencing and DEGs Analysis

**Figure 5:**
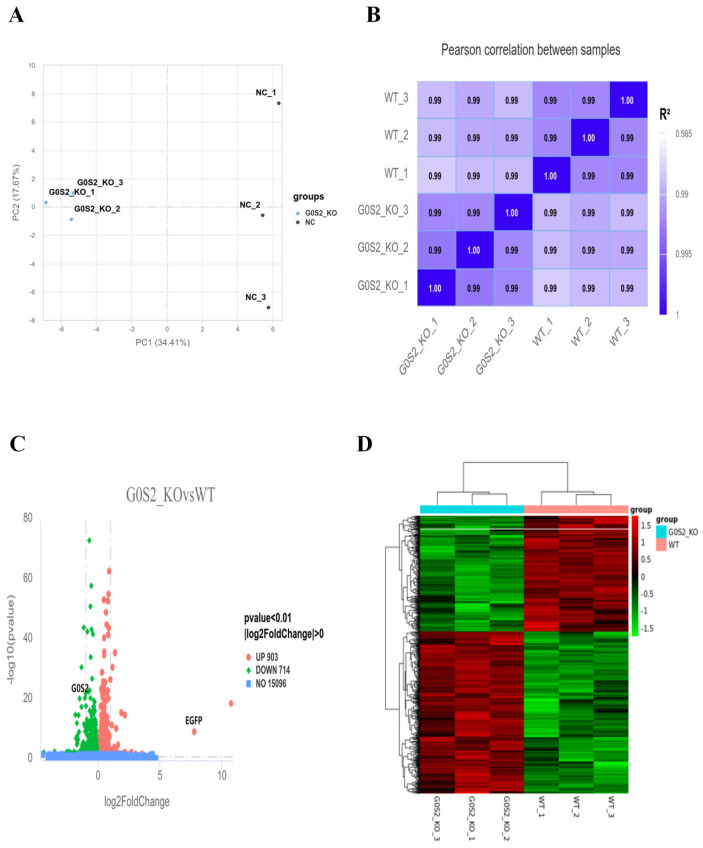
Transcriptome sequencing and DEG analysis. (**A**,**B**) PCA (**A**) and correlation heatmap (**B**) of the *G0S2*-KO and WT cell lines. (**C**,**D**) Volcano plot (**C**) and clustering heatmap (**D**) of differentially expressed genes (DEGs) between *G0S2*-KO and WT cell lines.

Transcriptome sequencing was conducted on *G0S2*-KO and WT cells (n = 3). Following RNA extraction and sequencing, the quality of the sequencing data was assessed and determined to satisfy the required standards. Principal component analysis (PCA) (Figure 5A) revealed a clear distinction between *G0S2*-KO and WT cells, with significant separation between the two groups. Stronger intra-group correlation than inter-group correlation was shown by values exceeding 0.99 in the Pearson correlation coefficients of gene expression levels across all samples (Figure 5B). Comparing *G0S2*-KO cells to WT cells, the analysis of gene expression revealed a total of 1617 differentially expressed genes (DEGs). Among them, 903 were upregulated, and 714 showed reduced expression (*p*-value < 0.01, |log2FoldChange| > 0). A thorough summary of the distribution of genes that are significantly differentially expressed between the two groups can be found in the volcano plot of DEGs (Figure 5C). Additionally, the successful creation of the *G0S2*-KO cell line was confirmed by the much higher EGFP expression and significantly lower *G0S2* expression in *G0S2*-KO cells as compared to WT cells. The differential gene clustering heatmap (Figure 5D) offers a clearer visualization of the gene expression differences between the two cell lines.

### 3.6. GO and KEGG Pathway Analysis

**Figure 6:**
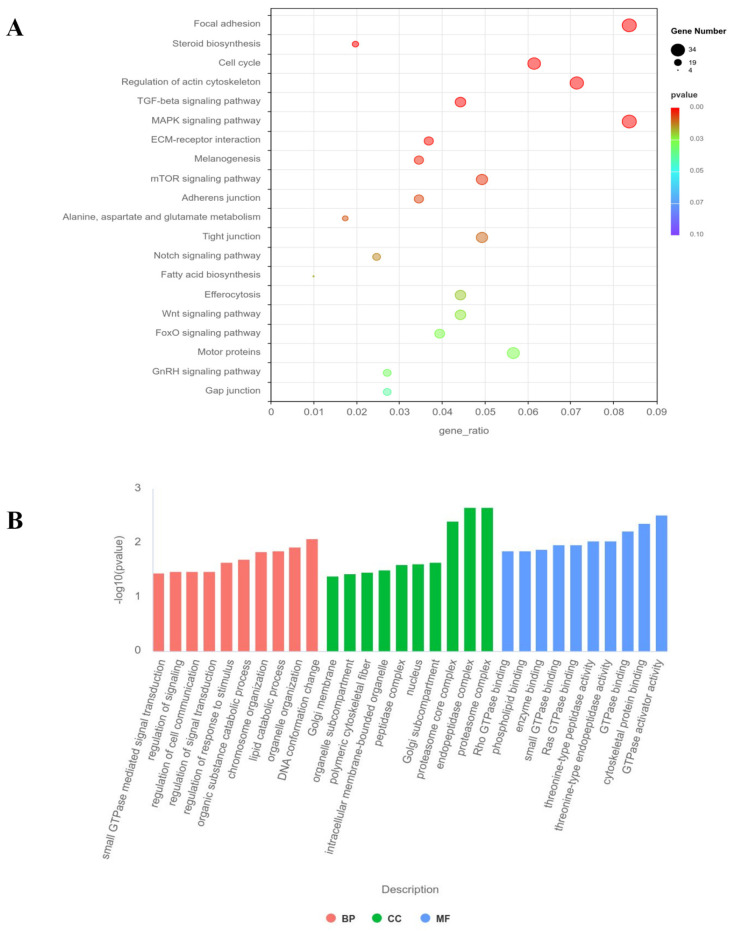
GO and KEGG pathway analysis. (**A**) KEGG pathway analysis, with the gene ratio on the X-axis and KEGG terms on the Y-axis. (**B**) GO pathway analysis, where the gene ratio is displayed on the Y-axis, and GO terms are plotted on the X-axis. Biological processes, cellular components, and molecular functions are represented by BP, CC, and MF, respectively.

GO enrichment analysis (Figure 6A) revealed that the differentially expressed genes in *G0S2*-KO cells are primarily enriched in core biological processes, including small GTPase signaling, cytoskeleton dynamics regulation, chromatin organization, and epigenetic regulation. Molecular function enrichment analysis suggested that these genes may be involved in the regulation of GTPase activity, kinase activity, and phospholipid metabolism. Cellular component enrichment results indicated that the differentially expressed genes are mainly concentrated in key cellular structures, including the cytoskeleton, chromatin, and Golgi apparatus. KEGG analysis (Figure 6B) showed that the differentially expressed genes in *G0S2*-KO cells are significantly enriched in metabolic regulatory pathways, such as fatty acid biosynthesis and sterol biosynthesis, as well as signaling pathways, related to the cell cycle and differentiation (e.g., mTOR, TGF-β, and MAPK pathways). Additionally, the differentially expressed genes are significantly enriched in pathways such as efferocytosis (apoptotic cell clearance), cytoskeleton dynamics regulation, and focal adhesion.

## 4. Discussion

This study demonstrates that the knockout of *G0S2* notably enhances the proliferation and apoptosis of chicken preadipocytes, while simultaneously inhibiting their differentiation. These results highlight the important function of *G0S2* in controlling chicken preadipocyte growth and development.

Prior investigations have suggested that *G0S2* helps maintain the quiescent state of cells by suppressing cell proliferation in human leukemia cells and hematopoietic stem cells [27,28]. In contrast, our results show that *G0S2* knockout causes enhanced DNA synthesis, increased cell viability, and accelerated progression from the G1 to the S phase. These observations imply that *G0S2* serves to inhibit cell proliferation in chicken preadipocytes, aligning with prior research indicating its function in limiting cell proliferation.

The role of *G0S2* in apoptosis regulation remains a topic of debate. Initial studies indicated that *G0S2* induces apoptosis by directly interacting with *Bcl-2*, blocking the development of protective *Bcl-2/Bax* heterodimers [16]. However, other research has suggested that *G0S2* reduces the production of reactive oxygen species (ROS) and inhibits apoptosis in endothelial cells, exerting a protective effect [17]. In this study, we found that *G0S2* knockout had a minor effect on the overall apoptosis of chicken preadipocytes, with the primary difference observed in early apoptotic cells. qPCR analysis showed that *G0S2* knockout significantly upregulated the expression of key apoptosis-related genes, such as caspase-3 and Fas. Western blot analysis indicated that the cleaved caspase-3 level increased in *G0S2* knockout cells compared to wild-type cells, although the increase was not particularly significant. These results suggest that *G0S2* knockout promotes apoptosis in chicken preadipocytes, but the pro-apoptotic effect is relatively mild, mainly reflected in the increased proportion of early apoptotic cells, with minimal impact on late apoptosis. These results corroborate earlier findings that silencing *G0S2* induces apoptosis in 3T3-L1 cells [29]. This indicates that *G0S2* plays an inhibitory role in apoptosis in chicken preadipocytes, highlighting its context-dependent regulatory effects across different cell types.

Additionally, *G0S2* is crucial in the regulation of adipocyte differentiation. It has been shown that the *G0S2* promoter contains potential *PPARγ* response elements, and *G0S2* is directly regulated by *PPARγ* [15]. Choi et al. demonstrated that overexpression of *G0S2* enhances differentiation in mouse 3T3-L1 preadipocytes, along with increased expression of *C/EBPα* and *PPARγ*. Conversely, *G0S2* knockdown suppresses adipocyte differentiation and decreases the expression of these key transcription factors [29]. Our data show that *G0S2*-KO cells have a significantly lower accumulation of intracellular lipid droplets compared to WT cells. In line with these findings, RT-qPCR analysis revealed that, after 72 h of differentiation, mRNA levels of *PPARγ*, *FASN*, *LPL*, *PLIN1*, and *C/EBPb* were markedly reduced in *G0S2*-KO cells relative to WT cells. These findings are consistent with previous observations that G0S2 deletion in chickens results in reduced abdominal fat formation [19], implying that G0S2 enhances differentiation and lipid droplet formation in chicken preadipocytes.

We used transcriptome sequencing on *G0S2*-KO cells and WT cells to learn more about the molecular processes controlling the growth, development, and function of *G0S2* in chicken preadipocytes. KEGG pathway analysis revealed that *G0S2* knockout significantly impacts several key signaling pathways, such as the cell cycle, MAPK, Notch, FOXO, and Wnt pathways. The MAPK pathway is well-established for its role in activating cyclin D transcription [30], and alterations in the cell cycle are known to influence cell proliferation [31,32]. Activation of Wnt and Notch signaling pathways has been shown to inhibit adipocyte differentiation in cultured cells [33,34], while the FOXO pathway promotes expression of the pro-apoptotic protein Bim, leading to apoptosis in endothelial progenitor cells [35]. These findings further suggest that *G0S2* may regulate the proliferation, differentiation, and apoptosis of chicken preadipocytes via these signaling pathways.

## 5. Conclusions

In conclusion, this study successfully generated a *G0S2* knockout chicken preadipocyte cell line using CRISPR/Cas9 technology. The results demonstrated that *G0S2* knockout significantly promoted the proliferation and apoptosis of chicken preadipocytes while inhibiting their differentiation. Transcriptome analysis revealed that *G0S2* knockout affected key signaling pathways, including the cell cycle, MAPK, Notch, FOXO, and Wnt pathways. The findings of this study contribute to understanding the function of the *G0S2* gene in chicken preadipocytes and lay a theoretical foundation for potential strategies aimed at controlling excessive fat deposition in chickens.

## Figures and Tables

**Table 1:** Oligonucleotide sequences employed for constructing G0S2 gRNA.

Name	Oligonucleotide Sequence (5′-3′)
G0S2-1e-sgRNA1	GCGTTCTTCGGCGTGGTCATCGG
G0S2-1e-sgRNA2	CTTCGGCGTGGTCATCGGTCTGG
G0S2-1e-sgRNA3	CGGCGTGGTCATCGGTCTGGTGG

**Table 2:** PCR detection primers.

Primer Name	Primer Sequences
G0S2-F	5′-AGAAGCCCAACAGGAAGATG-3′
G0S2-R	5′-CTTGCTCTGCTCCAACACC-3′
EGFP-F	5′-CCAGCAGAACACCCCC-3′
EGFP-R	5′-CTCGTCCATGCCGAGA-3′
PPARγ-F	5′-GTGCAATCAAAATGGAGCC-3′
PPARγ-R	5′-CTTACAACCTTCACATGCA-3′
LPL-F	5′-ATGCTGATGCCCCTATC -3′
LPL-R	5′-TTCTGAATCCCAATGCT-3′
FASN-F	5′-ATGGGTATTGTCGCTCT-3′
FASN-R	5′-CACCTTGCTCCTTAAAG-3′
C/EBPβ-F	5′-ACCTGTCCACCTCGTCC-3′
C/EBPβ-R	5′-GCAGCCTCTCGTTCTCG-3′
PLIN1-F	5′-GGGGTGACTGGCGGTTGTA-3′
PLIN1R	5′-GCCGTAGAGGTTGGCGTAG-3′
β-actin-F	5′-CCAGCCATCTTTCTTGGGTA-3′
β-actin-R	5′-ATGCCAGGGTACATTGTGGT-3′
caspas3-F	5′-ATAAGAACTTCCACCGA-3′
caspas3-R	5′-GCAACACACAAACAAAA-3′
FAS-F	5′-AGATGTTGACCTGACCC-3′
FAS-R	5′-CTCCCATTCCATGTTTT-3′
PCNA-F	5′-CGTTGGCTCTAGTGTTT-3′
PCNA-R	5′-GCTTCTTCCTCTTTGTC-3′
CCNDA-F	5′-CTTGGATGCTGGAGGTC-3′
CCNDA-R	5′-GCTTTTCTTGAGGGGTT-3′

## Data Availability

The data presented in this study are available on request from the corresponding author. The data are not publicly available due to privacy restrictions and the long extension of datasets.
